# Knowledge, Attitudes, and Clinical Preparedness of Dentists for Medical Emergencies: A Nationwide Cross-Sectional Survey

**DOI:** 10.3390/healthcare14060732

**Published:** 2026-03-13

**Authors:** Suzan Cangül, Makbule Taşyürek, Özkan Adıgüzel, Fırat Aşır

**Affiliations:** 1 Department of Restorative Dentistry, Faculty of Dentistry, Dicle University, Diyarbakır 21280, Türkiye; 2 Department of Endodontics, Faculty of Dentistry, Dicle University, Diyarbakır 21280, Türkiye; 3 Department of Histology and Embryology, Faculty of Medicine, Dicle University, Diyarbakır 21280, Türkiye; firatasir@gmail.com

**Keywords:** medical emergencies, dental practice, emergency preparedness, dentists’ knowledge, patient safety, continuing professional education

## Abstract

**Background**: Medical emergencies in dental practice are uncommon but may have serious consequences if not promptly recognized and managed. Dentists are expected to identify and initiate appropriate interventions during such events; however, the extent to which theoretical knowledge translates into clinical confidence and preparedness remains unclear. **Methods**: This nationwide cross-sectional survey evaluated dentists’ knowledge, attitudes, and preparedness regarding medical emergencies encountered in routine dental practice. A total of 300 dentists practicing in Türkiye completed two structured questionnaires: a scenario-based single-best-answer multiple-choice questionnaire assessing knowledge of medical emergencies and a Likert-scale questionnaire evaluating attitudes and clinical preparedness. Of the 450 dentists invited to participate, 300 completed the survey (response rate: 66.6%). Overall knowledge scores were calculated from 16 emergency scenarios, and participants were categorized into knowledge-level groups. Associations between knowledge, attitudes, and availability of emergency resources were analyzed using chi-square tests with effect size estimation. **Results**: The median overall knowledge score was 11 (IQR: 9–13). While high correct response rates were observed for commonly encountered emergencies such as syncope and intraoral bleeding, lower accuracy was noted for high-risk conditions including hypertensive crisis, anaphylaxis, and epileptic seizures. Only 40% of dentists reported feeling sufficiently competent to manage medical emergencies, and avoidance of treating high-risk patients was common. Higher knowledge levels and availability of emergency equipment and medications were significantly associated with greater self-perceived competence and reduced avoidance behavior. **Conclusions**: Although dentists demonstrate adequate theoretical knowledge of medical emergencies, significant gaps persist in clinical confidence, preparedness, and management of high-risk scenarios. Strengthening emergency preparedness in dental practice requires structured, hands-on training and improved access to essential emergency resources to ensure patient safety and support effective clinical decision-making.

## 1. Introduction

Medical emergencies in dental practice represent rare but potentially life-threatening events that require prompt recognition and immediate intervention [1]. Advances in healthcare and increased life expectancy have resulted in a growing population of dental patients with complex systemic conditions, including cardiovascular disease, diabetes mellitus, and respiratory disorders [2,3]. In addition, stress, anxiety, and the use of local anesthetics during dental procedures may independently trigger acute medical events, even in individuals without known systemic disease [4,5]. Consequently, dental clinics have increasingly become settings in which medical emergencies may arise unexpectedly, placing a critical responsibility on dentists to ensure patient safety [6].

Despite the relatively low frequency of severe emergencies, their consequences can be substantial when early recognition or appropriate management is delayed [7]. Previous studies have shown that most medical emergencies encountered in dental settings are preventable or manageable with timely first-line interventions [8,9]. However, effective emergency management requires more than theoretical knowledge alone; it depends on clinical preparedness, availability of appropriate equipment and medications, and the clinician’s confidence in initiating emergency care [10]. Deficiencies in any of these components may compromise patient outcomes and increase medico-legal risk [11].

The existing literature has largely focused on isolated aspects of emergency management in dentistry, such as dentists’ theoretical knowledge, the availability of emergency drugs and equipment, or attitudes toward treating medically compromised patients [12]. Many studies have been conducted in limited geographic regions or among dental students rather than actively practicing clinicians [6,13]. As a result, there remains a gap in understanding how knowledge, attitudes, and real-world preparedness coexist and interact within routine dental practice, particularly at a national level.

Undergraduate training in the management of medical emergencies may vary considerably across countries and dental education systems [11]. These differences often relate to the level of curricular emphasis, the amount of theoretical teaching and practical exposure, and the allocation of curricular credits or ECTS within dental programs [14,15]. As a result, dentists may graduate with heterogeneous levels of baseline knowledge, clinical confidence, and preparedness to manage medical emergencies in routine practice.

A comprehensive evaluation of dentists’ readiness to manage medical emergencies is essential to identify critical gaps that may affect patient safety [16]. Understanding not only what dentists know but also how confident they feel, how well equipped their clinics are, and how these factors interact in daily practice can provide valuable insight for improving emergency preparedness. Therefore, the aim of this nationwide cross-sectional study was to provide an integrated assessment of dentists’ knowledge, attitudes, and clinical preparedness regarding medical emergencies in dental practice and to examine the associations between knowledge levels, self-perceived competence, and the availability of emergency equipment and medications.

## 2. Materials and Methods

### 2.1. Study Design and Ethical Approval

This study was designed as a nationwide, cross-sectional, questionnaire-based survey conducted to evaluate dentists’ knowledge, attitudes, and preparedness regarding medical emergencies encountered in routine dental practice. The study protocol was reviewed and approved by the Ethics Committee of the Faculty of Dentistry, Dicle University (Decision No: 2020-16; Date: 27 May 2020). All procedures were carried out in accordance with the principles of the Declaration of Helsinki. Written informed consent was obtained from all participants prior to their inclusion in the study. Participation was voluntary, and all responses were collected anonymously to ensure confidentiality.

### 2.2. Study Population and Sampling

Dentists were recruited using a convenience sampling approach based on the list of active dentists obtained from the Turkish Dental Association. Invitations were distributed electronically via e-mail or administered face-to-face by a volunteer dentist. Participation was voluntary, and dentists who met the inclusion criteria were invited to complete the questionnaire. Because the study aimed to capture responses from dentists practicing in different clinical environments across Türkiye, both electronic and face-to-face data collection methods were used to increase participation.

A total of 450 dentists were invited to participate in the survey. Of these, 300 dentists completed the questionnaire, yielding a response rate of 66.6%. The questionnaire was distributed either electronically via e-mail or administered face-to-face by a volunteer dentist who was not involved in the study design, data analysis, or publication process. Each participant completed the survey only once. No personal identifiers, including names or institutional affiliations, were recorded or accessible to the researchers. However, limited background information was collected from participants, including age group, gender, years of clinical experience, professional status (general dentist or specialist), practice setting, and previous emergency training. Data collection was carried out between 2 October 2023 and 15 December 2024. During this period, no national policy changes or major guideline updates regarding the management of medical emergencies in dental practice were identified that could systematically influence participants’ responses.

### 2.3. Sample Size Calculation

Sample size calculation was performed using G*Power software version 3.1 (Heinrich Heine University, Düsseldorf, Germany). Based on a previously reported proportion (*p* = 0.46) from the literature, the minimum required sample size was calculated as 297 participants using a one-sample binomial test with a significance level (α) of 0.05 and a statistical power of 0.95. The final sample size of 300 participants met and slightly exceeded this requirement.

### 2.4. Survey Instrument

The survey instrument consisted of two structured questionnaires designed to evaluate dentists’ knowledge and preparedness regarding medical emergencies in dental practice. The first questionnaire included 16 scenario-based multiple-choice questions assessing knowledge of diagnosis and initial management of medical emergencies that may occur during dental treatment.

The second questionnaire consisted of five statements evaluating dentists’ attitudes and self-perceived preparedness regarding medical emergencies. The statements were as follows:

(1) “I avoid treating patients at risk of developing a medical emergency.”
(2) “I think I have sufficient knowledge and experience related to medical emergencies and the required interventions.”
(3) “In the clinic where I work, emergency equipment is available.”
(4) “In the clinic where I work, emergency medications are available.”
(5) “I am willing to participate in training programs related to medical emergencies and their management.”

Responses to these statements were recorded using a five-point Likert scale ranging from “strongly agree” to “strongly disagree”. The questionnaire was developed by the research team based on commonly reported medical emergency scenarios in dental practice and relevant literature. Prior to the main study, a pilot study was conducted to evaluate the clarity and internal consistency of the instrument. All returned questionnaires were complete, and no missing data were identified for the variables included in the analysis.

### 2.5. Knowledge-Based Questionnaire

The first questionnaire comprised 16 multiple-choice questions designed to assess dentists’ knowledge of diagnosis and initial management of common medical emergencies that may arise during dental treatment. The questions were scenario-based and covered a range of emergency conditions, including hypoglycemia, syncope, orthostatic hypotension, foreign body aspiration, asthma attack, epileptic seizure, hypertensive crisis, allergic reactions, anaphylaxis, acute coronary syndrome, cerebrovascular events, anxiety-related hyperventilation, and intraoral bleeding. Each question included one correct response and two incorrect alternatives.

### 2.6. Attitudes and Preparedness Questionnaire

The second questionnaire consisted of five statements aimed at evaluating dentists’ self-perceived preparedness, attitudes toward treating patients at risk of medical emergencies, availability of emergency equipment and medications in the clinical setting, and willingness to participate in emergency-related training programs. Responses were recorded using a five-point Likert scale ranging from “strongly agree” to “strongly disagree”.

### 2.7. Validity and Reliability

Prior to the main data collection, the questionnaire was pilot-tested on a group of 50 dentists to assess clarity, comprehensibility, and potential ambiguities in the survey items. Feedback from the pilot group indicated that all items were clear and understandable; therefore, no modifications were made. The results of the pilot study were not included in the final analysis. Content validity was evaluated by five academic dentists with expertise in their respective fields. The internal consistency of the five Likert-scale items assessing attitudes and preparedness was evaluated using Cronbach’s alpha (α = 0.82).

### 2.8. Data Collection Procedure

Questionnaires were completed anonymously either via electronic submission or face-to-face administration. In face-to-face settings, the volunteer dentist ensured that inclusion criteria were met and that each participant completed the questionnaire independently. The researchers had access only to anonymized datasets and were not involved in the questionnaire administration process. This approach minimized potential response bias and ensured participant confidentiality.

### 2.9. Statistical Analysis

Statistical analysis was performed using SPSS software version 25.0 (IBM Corp., Armonk, NY, USA). Descriptive statistics were used to summarize the data and were presented as number (*n*), percentage (%), and 95% confidence intervals where appropriate. Responses to the knowledge-based questionnaire were evaluated according to a predefined answer key. Comparisons between categorical variables were conducted using the chi-square (χ^2^) test. Effect sizes were reported using Cramér’s V to provide an estimate of the strength of associations. A two-sided *p*-value of <0.05 was considered statistically significant.

## 3. Results

### 3.1. Participant Characteristics

The demographic and professional characteristics of the participating dentists are presented in Table 1. A total of 300 dentists participated in the study. The largest proportion of participants was aged between 25 and 34 years (42.0%), and females constituted 56.0% of the sample. Most participants had between 1 and 10 years of clinical experience (62.0%). General dentists accounted for 72.0% of respondents, while 28.0% were specialists. A majority of dentists worked in private practice settings (63.0%). Slightly more than half of the participants (54.0%) reported having received previous training related to medical emergencies, while 49.0% indicated that they held a basic life support (BLS) certification.

### 3.2. Knowledge-Based Medical Emergency Scenarios

Correct and incorrect response distributions for the sixteen scenario-based medical emergency questions are presented in Table 2. Overall, dentists demonstrated high accuracy in the management of frequently encountered emergencies. The highest correct response rates were observed for the management of syncope, with 291 dentists (97.0%) correctly selecting placement of the patient in the Modified Trendelenburg position, and for severe intraoral bleeding, where 288 dentists (96.0%) selected tamponade application as the appropriate intervention. High levels of correct responses were also observed for asthma attacks (276 dentists, 92.0%) and hypersensitivity reactions such as urticaria following nonsteroidal anti-inflammatory drug use (270 dentists, 90.0%). Moderate levels of accuracy were recorded for several other emergency scenarios. Orthostatic hypotension was correctly identified and managed by 252 dentists (84.0%), while foreign body aspiration requiring the Heimlich maneuver was correctly identified by 219 dentists (73.0%). Similar response rates were observed for acute chest pain suggestive of acute coronary syndrome (219 dentists, 73.0%) and cerebrovascular events (216 dentists, 72.0%). Management of severe hypoglycemia with intravenous dextrose was correctly identified by 213 dentists (71.0%), and 198 dentists (66.0%) correctly indicated that the Heimlich maneuver should be continued until assistance arrives. Lower correct response rates were observed for more complex or less frequently encountered emergencies. Anaphylaxis requiring epinephrine administration was correctly identified by 198 dentists (66.0%), while the appropriate first approach to an epileptic seizure was correctly identified by 186 dentists (62.0%). Hyperventilation related to anxiety was correctly diagnosed and managed by 174 dentists (58.0%), and hypertensive crisis requiring pharmacologic intervention was correctly identified by 156 dentists (52.0%). Overall, these findings indicate that dentists demonstrate stronger knowledge in the management of common and frequently encountered emergencies, whereas lower accuracy rates are observed for more complex, high-risk, or pharmacologically demanding emergency situations.

### 3.3. Attitudes and Preparedness Regarding Medical Emergencies

Dentists’ attitudes toward medical emergencies and their self-reported preparedness are presented in Table 3. For the statement “I avoid treating patients at risk of developing a medical emergency,” 10% of participants strongly agreed and 26% agreed, whereas 28% disagreed and 9% strongly disagreed. Regarding self-perceived competence in managing medical emergencies, 7% strongly agreed and 28% agreed that they felt sufficiently competent, while 27% disagreed and 5% strongly disagreed. Emergency equipment and emergency medications were reported as available by 52% and 52% of dentists, respectively, when combining strongly agree and agree responses. A large majority of participants (81%) indicated willingness to participate in training programs related to medical emergencies.

### 3.4. Association Between Knowledge Level and Preparedness (Additional Analysis)

Additional analyses demonstrated significant associations between knowledge level and several preparedness-related outcomes (Table 4). Knowledge level was significantly associated with self-perceived competence in managing medical emergencies (χ^2^(4) = 18.6, *p* < 0.001; Cramér’s V = 0.25). In addition, knowledge level was associated with avoidance of treating patients at risk of medical emergencies (χ^2^(4) = 12.9, *p* = 0.012; Cramér’s V = 0.21). Availability of emergency equipment in the clinical setting was associated with a lower tendency to avoid treating high-risk patients (χ^2^(2) = 10.8, *p* = 0.004; Cramér’s V = 0.19). A similar association was observed for the availability of emergency medications (χ^2^(2) = 9.6, *p* = 0.008; Cramér’s V = 0.18).

## 4. Discussion

Medical emergencies in dental practice represent a low-frequency but high-impact challenge that directly affects patient safety and clinical outcomes [17]. The present nationwide survey provides an integrated assessment of dentists’ theoretical knowledge, self-perceived competence, and clinical preparedness, offering insight into how these components interact in real-world dental settings. Beyond the descriptive findings, the results reveal structural and educational gaps that help explain why adverse outcomes may still occur despite acceptable levels of theoretical knowledge.

One of the most notable findings of this study is the disparity between dentists’ performance in managing common versus high-risk medical emergencies. Conditions such as syncope, severe intraoral bleeding, and asthma attacks were associated with very high correct response rates, a pattern consistently reported in the literature [18]. Previous surveys from Europe, the Middle East, and Asia have similarly shown that dentists demonstrate greater confidence and accuracy in emergencies that are frequently encountered and managed conservatively, often without the need for advanced pharmacological intervention or invasive procedures [11,18]. This suggests that repeated exposure and experiential learning play a critical role in shaping clinical competence in dental emergencies.

In contrast, substantially lower accuracy rates were observed for hypertensive crisis, anaphylaxis, epileptic seizures, and anxiety-related hyperventilation [19,20]. These conditions are clinically more complex, may progress rapidly, and often require immediate pharmacological or systemic intervention [21]. The relatively poor performance in these scenarios is consistent with earlier studies reporting limited confidence among dentists in administering emergency drugs, managing cardiovascular instability, or differentiating between neurologic and psychogenic conditions [22]. Importantly, these emergencies, although less frequent, are among those most likely to result in severe morbidity or mortality if mismanaged, underscoring their disproportionate clinical significance [10]. In addition to individual training gaps, structural factors within the educational and professional system may also contribute to these findings. Variability in undergraduate dental curricula regarding medical emergency management and the absence of standardized mandatory postgraduate training programs may lead to differences in dentists’ preparedness levels. The findings of the present study may partly reflect the variability in undergraduate dental education and the absence of universally standardized postgraduate training in medical emergency management. Differences in curricular structure, including the balance between theoretical instruction and practical training, may contribute to variations in dentists’ preparedness when confronted with emergency situations. These observations highlight the importance of developing more standardized educational frameworks that integrate both theoretical knowledge and practical emergency training during dental education.

The overall knowledge score analysis further clarifies this issue. While the median score indicates a generally acceptable level of cognitive knowledge, categorization into knowledge levels reveals that a substantial proportion of dentists fall within the moderate or low knowledge groups. This finding aligns with prior reports suggesting that dental education often emphasizes recognition of emergencies but provides insufficient depth in advanced decision-making and pharmacological management [23,24]. Moreover, knowledge assessments alone may overestimate readiness if not complemented by evaluation of confidence and practical preparedness [25].

A critical contribution of the present study is the demonstration of a clear disconnect between knowledge and self-perceived competence. Despite nearly one-third of participants being classified within the high knowledge category, fewer than half reported feeling sufficiently competent to manage medical emergencies. This phenomenon has been described previously and reflects a well-recognized educational gap between theoretical instruction and applied clinical skills. In high-stress situations such as medical emergencies, uncertainty and lack of hands-on experience may override cognitive knowledge, leading to hesitation, delayed intervention, or avoidance behaviors [26,27].

Avoidance of treating patients at risk of medical emergencies represents an especially important clinical and ethical concern [28]. In the present study, avoidance behavior was not uncommon and was significantly associated with both lower knowledge levels and lack of emergency resources. Similar findings have been reported internationally, where dentists often prefer referral or postponement when confronted with medically complex patients [29]. While such caution may be appropriate in selected cases, systematic avoidance can contribute to delayed care, increased healthcare burden, and potential inequities in access to dental treatment for medically compromised patients [30,31].

The association between the availability of emergency equipment and medications and reduced avoidance behavior highlights the systemic dimension of preparedness [32]. Dentists practicing in clinics equipped with essential emergency resources were significantly less likely to report avoiding the treatment of high-risk patients, suggesting that preparedness is not solely an individual responsibility but also an organizational one [33,34]. Inadequate infrastructure may reinforce anxiety, amplify perceived risk, and limit dentists’ willingness to intervene decisively. International guidelines consistently emphasize that emergency preparedness in dental settings requires both trained personnel and immediate access to appropriate drugs and equipment; failure in either domain undermines effective emergency response [35].

Encouragingly, the high willingness of dentists to participate in emergency-related training programs indicates strong professional awareness of existing limitations [36]. This finding mirrors reports from multiple regions and suggests that dentists recognize the need for ongoing education beyond undergraduate training. Importantly, the data support a shift from predominantly theoretical instruction toward practical, simulation-based, and scenario-driven training models [37]. Evidence from medical and dental education demonstrates that repeated simulation exposure improves retention, confidence, and performance during real emergencies, particularly for low-frequency, high-risk events [38,39].

Several limitations should be considered when interpreting the findings of this study. First, the data were based on self-reported responses, which may be subject to reporting or recall bias. In addition, responses may have been influenced by social desirability bias, as participants might have tended to report higher levels of knowledge, preparedness, or confidence than their actual clinical practice. Second, although basic demographic information was collected, more detailed demographic variables were not available, which limits the ability to explore subgroup differences in greater depth. Third, participation in the survey was voluntary, and therefore selection or non-response bias cannot be excluded; dentists with greater interest or confidence in managing medical emergencies may have been more likely to participate. These factors should be considered when interpreting the generalizability of the results. In addition, although the survey aimed to include dentists practicing in different clinical settings across Türkiye, the geographic distribution of participants was not systematically controlled. Furthermore, the use of both electronic and face-to-face survey administration methods may have introduced minor response differences, although the questionnaire content and procedures were identical for all participants.

## 5. Conclusions

Medical emergencies in dental practice are uncommon but potentially life-threatening events that require rapid recognition and effective initial management. The findings of this nationwide survey indicate that while dentists generally possess adequate theoretical knowledge, important gaps remain in clinical confidence, preparedness, and management of high-risk emergency scenarios. Knowledge level and the availability of emergency equipment and medications were associated with dentists’ perceived competence and their willingness to treat medically high-risk patients. These results highlight the need to strengthen emergency preparedness in dental practice through more structured, hands-on training and improved access to essential emergency resources. Practical training approaches, such as simulation-based emergency drills, periodic verification of emergency equipment and medications in dental clinics, and structured continuing education programs focusing on the practical management of high-risk medical emergencies may help bridge the gap between theoretical knowledge and clinical readiness, ultimately contributing to safer dental care. Improving emergency preparedness in dental practice should begin at the undergraduate level through more standardized curricular frameworks and the stronger integration of simulation-based emergency training within dental education.

## Figures and Tables

**Table 1:** Demographic and professional characteristics of participating dentists (*n* = 300).

Variable	Category	*n* (%)
Age (years)	25–34	126 (42.0%)
	35–44	90 (30.0%)
	45–54	54 (18.0%)
	≥55	30 (10.0%)
Gender	Male	132 (44.0%)
	Female	168 (56.0%)
Years of clinical experience	1–5 years	102 (34.0%)
	6–10 years	84 (28.0%)
	11–20 years	69 (23.0%)
	>20 years	45 (15.0%)
Professional status	General dentist	216 (72.0%)
	Specialist	84 (28.0%)
Practice setting	Private practice	189 (63.0%)
	Public hospital/university clinic	111 (37.0%)
Previous emergency training	Yes	162 (54.0%)
	No	138 (46.0%)
Basic life support (BLS) certification	Yes	147 (49.0%)
	No	153 (51.0%)

**Table 2:** Dentists’ responses to scenario-based questions evaluating knowledge of medical emergency management in dental practice (*n* = 300).

Medical Emergency Scenario	Response Option	*n* (%)	95% CI
Patient with sweating, irritability, and hand tremor	Hypertension	36 (12%)	8.6–16.2
	Conversion disorder	81 (27%)	22.1–32.4
	**Hypoglycemia**	**183 (61%)**	**55.2–66.6**
Foreign body aspiration with cyanosis and inability to speak	Place patient on left side	30 (10%)	6.8–14.0
	Strike patient on back	51 (17%)	12.9–21.7
	**Perform Heimlich manoeuvre**	**219 (73%)**	**67.6–77.9**
Dizziness and pallor after treatment	Epileptic seizure	21 (7%)	4.4–10.5
	**Orthostatic hypotension + supine position**	**252 (84%)**	**79.3–88.0**
	Conversion	27 (9%)	6.0–13.0
Syncope during treatment	Intravenous isotonic	3 (1%)	0.2–2.9
	Give fruit juice	6 (2%)	0.7–4.3
	**Modified Trendelenburg position**	**291 (97%)**	**94.4–98.7**
Skin rash after NSAID use	Angioedema + C1 inhibitor	9 (3%)	1.4–5.6
	**Urticaria + antihistamine**	**270 (90%)**	**86.0–93.2**
	Anaphylaxis + epinephrine	21 (7%)	4.4–10.5
Epileptic seizure during treatment	**Prevent patient injury**	**186 (62%)**	**56.2–67.5**
	Open jaws	66 (22%)	17.4–27.3
	Fixate extremities	48 (16%)	12.1–20.6
Hypertensive crisis (BP 180/100 mmHg)	**Oral captopril**	**156 (52%)**	**46.2–57.8**
	Referral to cardiologist	66 (22%)	17.4–27.3
	Repeat regular medication	78 (26%)	21.1–31.5
Severe intraoral bleeding	**Apply tamponade**	**288 (96%)**	**93.0–98.0**
	Lean patient forward	3 (1%)	0.2–2.9
	Intravenous isotonic	9 (3%)	1.4–5.6
Sudden collapse without cyanosis	**Anxiety disorder**	**165 (55%)**	**49.2–60.7**
	Conversion	114 (38%)	32.4–43.8
	Syncope	21 (7%)	4.4–10.5
Asthma attack	**Oxygen + short-acting β2 agonist**	**276 (92%)**	**88.3–95.0**
	Long-acting β2 agonist	15 (5%)	2.8–8.1
	IV antihistamine + steroid	9 (3%)	1.4–5.6
Acute chest pain	IV isotonic + call emergency	48 (16%)	12.0–20.6
	**Oxygen + ASA + call emergency**	**219 (73%)**	**67.6–77.9**
	Immediate ED referral	33 (11%)	7.7–15.1
Hypoglycemia with unconsciousness	Intravenous isotonic	36 (12%)	8.5–16.2
	Fruit juice	51 (17%)	12.9–21.7
	**IV dextrose**	**213 (71%)**	**65.4–76.1**
Duration of Heimlich manoeuvre	**Until assistance arrives**	**198 (66%)**	**60.3–71.4**
	15 min	84 (28%)	23.0–33.6
	45 min	18 (6%)	3.6–9.3
Stroke symptoms	Epileptic seizure	63 (21%)	16.7–26.1
	**Cerebrovascular event**	**216 (72%)**	**66.5–77.1**
	Acute coronary syndrome	21 (7%)	4.4–10.5
Anaphylaxis symptoms	**Drug allergy + antihistamine**	42 (14%)	10.3–18.5
	**Anaphylaxis + epinephrine**	**198 (66%)**	**60.3–71.4**
	Anaphylaxis + atropine	60 (20%)	15.6–25.0
Hyperventilation	Conversion	102 (34%)	28.7–39.6
	**Hyperventilation + controlled breathing**	**174 (58%)**	**52.2–63.6**
	Epileptic seizure	24 (8%)	5.2–11.7

Correct responses are shown in bold. CI: confidence interval.

**Table 3:** Dentists’ attitudes and preparedness regarding medical emergencies (five-point Likert distribution).

Statement	Strongly Agree *n* (%)	Agree *n* (%)	Neutral *n* (%)	Disagree *n* (%)	Strongly Disagree *n* (%)
Avoid treating high-risk patients	30 (10.0)	78 (26.0)	81 (27.0)	84 (28.0)	27 (9.0)
Feel sufficiently competent	21 (7.0)	84 (28.0)	99 (33.0)	81 (27.0)	15 (5.0)
Emergency equipment available	42 (14.0)	114 (38.0)	42 (14.0)	54 (18.0)	48 (16.0)
Emergency drugs available	39 (13.0)	117 (39.0)	45 (15.0)	54 (18.0)	45 (15.0)
Willing to participate in emergency training	117 (39.0)	126 (42.0)	39 (13.0)	15 (5.0)	3 (1.0)

**Table 4:** Associations between knowledge level and preparedness (χ^2^ analyses).

Outcome	Predictor	χ^2^ (df)	*p*-Value	Cramér’s V
Self-perceived competence	Knowledge level (Low–Moderate–High)	18.6 (4)	<0.001	0.25
Avoid treating high-risk patients	Knowledge level	12.9 (4)	0.012	0.21
Avoid treating high-risk patients	Emergency equipment available (Yes/No)	10.8 (2)	0.004	0.19
Avoid treating high-risk patients	Emergency drugs available (Yes/No)	9.6 (2)	0.008	0.18

## Data Availability

The data presented in this study are available upon request from the corresponding author. The data are not publicly available due to patient privacy.
